# Service evaluation of an educational intervention to improve sexual health services in primary care implemented using a step-wedge design: analysis of chlamydia testing and diagnosis rate changes

**DOI:** 10.1186/s12889-016-3343-z

**Published:** 2016-08-02

**Authors:** Katy Town, Cliodna A. M. McNulty, Ellie J. Ricketts, Thomas Hartney, Anthony Nardone, Kate A. Folkard, Andre Charlett, J. Kevin Dunbar

**Affiliations:** 1 HIV/STI Department, National Infection Service, Public Health England, London, UK; 2 Public Health England Primary Care Unit, Microbiology Department, Gloucestershire Royal Hospital, Gloucester, UK; 3 Statistics, Modelling and Economics Department, Public Health England, 61 Colindale Avenue, NW9 5EQ London, UK

**Keywords:** Primary care, Chlamydia screening, HIV testing, Contraception, Condoms, Education, Service evaluation, Pilot, Step-wedge, Implementation

## Abstract

**Background:**

Providing sexual health services in primary care is an essential step towards universal provision. However they are not offered consistently. We conducted a national pilot of an educational intervention to improve staff’s skills and confidence to increase chlamydia testing rates and provide condoms with contraceptive information plus HIV testing according to national guidelines, known as 3Cs&HIV. The effectiveness of the pilot on chlamydia testing and diagnosis rates in general practice was evaluated.

**Methods:**

The pilot was implemented using a step-wedge design over three phases during 2013 and 2014 in England. The intervention combined educational workshops with posters, testing performance feedback and continuous support. Chlamydia testing and diagnosis rates in participating general practices during the control and intervention periods were compared adjusting for seasonal trends in chlamydia testing and differences in practice size. Intervention effect modification was assessed for the following general practice characteristics: chlamydia testing rate compared to national median, number of general practice staff employed, payment for chlamydia screening, practice urban/rurality classification, and proximity to sexual health clinics.

**Results:**

The 460 participating practices conducted 26,021 tests in the control period and 18,797 tests during the intervention period. Intention-to-treat analysis showed no change in the unadjusted median tests and diagnoses per month per practice after receiving training: 2.7 vs 2.7; 0.1 vs 0.1. Multivariable negative binomial regression analysis found no significant change in overall testing or diagnoses post-intervention (incidence rate ratio (IRR) 1.01, 95 % confidence interval (CI) 0.96–1.07, *P* = 0.72; 0.98 CI 0.84–1.15, *P* = 0.84, respectively). Stratified analysis showed testing increased significantly in practices where payments were in place prior to the intervention (IRR 2.12 CI 1.41–3.18, *P* < 0.001) and in practices with 6–15 staff (6–10 GPs IRR 1.35 (1.07–1.71), *P* = 0.012; 11–15 GPs IRR 1.37 (1.09–1.73), *P* = 0.007).

**Conclusion:**

This national pilot of short educational training sessions found no overall effect on chlamydia testing in primary care. However, in certain sub-groups chlamydia testing rates increased due to the intervention. This demonstrates the importance of piloting and evaluating any service improvement intervention to assess the impact before widespread implementation, and the need for detailed understanding of local services in order to select effective interventions.

**Electronic supplementary material:**

The online version of this article (doi:10.1186/s12889-016-3343-z) contains supplementary material, which is available to authorized users.

## Background

In England, diagnoses of sexually transmitted infections (STIs) are increasing. Young adults aged 16–25 continue to be at the highest risk of contracting an STI. Chlamydia, which can cause pelvic inflammatory disease and infertility, is the most commonly diagnosed infection in this age group with over 200,000 diagnoses made in 2013 and 2014 [1, 2]. Sexual health services have traditionally been provided in specialist services including genitourinary medicine clinics (GUM). However general practice has been identified by successive UK governments and national public health bodies as an important facilitator in the provision of sexual health services through increased testing, partner follow up and prevention [3, 4]. An estimated 303.9 million primary care consultations occur every year [5], and almost 75 % of young people attend their general practice annually [6]. General practice is an accessible and acceptable setting for patients’ to receive sexual health services [3, 4, 6–13] and the English national guidelines recommend general practices provide chlamydia tests to all sexually active <25 year olds [14].

Despite this, sexual health services are not universally offered in general practices, leaving missed opportunities to diagnose infections and provide contraceptives [15, 16]. A lack of education and training for all general practice staff, including nurses and receptionists, contribute to this shortfall in service [17, 18]. Complex multifaceted interventions to improve sexual health service provision in primary care have successfully improved the skills, confidence and motivation of practice staff to offer sexual health services to patients. These interventions consist of components including educational training sessions, promotional material, automated reminders, specific payments for chlamydia testing and testing rate feedback [19–28]. However, success has varied and these interventions may not work outside of trial conditions. Differences between a research setting and practical implementation may relate to funding, enthusiasm for the intervention and changes to policies and responsibilities for service delivery [29]. Therefore, there is a need to pilot and further evaluate interventions when translating research into practice.

The Chlamydia Intervention Randomised Trial (CIRT) increased chlamydia testing in general practices that received the intervention [19]. The CIRT intervention combined educational workshops with posters, testing performance feedback and on-going support from a researcher to significantly increase chlamydia testing rates in practices receiving the intervention. Public Health England (PHE) has expanded and piloted this intervention to incorporate policy changes since CIRT, such as the integration of chlamydia testing with other sexual and reproductive services. The expanded intervention (3Cs&HIV) encouraged general practice staff to routinely offer chlamydia testing, and provide information about the provision of contraceptive services and free condoms (the ‘3Cs’) to all 15–24 year olds regardless of the type of consultation, and offer HIV testing according to national guidelines. The aim of the pilot was to determine the feasibility of implementing this intervention outside of trial conditions with existing local authority funded staff. This paper presents part of the results of the service evaluation of this pilot, specifically the primary outcome of chlamydia testing and diagnostic rate changes in participating general practices. HIV testing rates and contraceptive advice provision will be evaluated separately. Process evaluation measures assessing uptake of the intervention are also presented.

## Methods

### Study design

The pilot protocol details the full methodology of this service evaluation including sample size determination and inclusion/exclusion criteria [30]. All local authorities (LAs) in England were invited to participate and provide nominated trainers. Trainers recruited general practices and delivered the two training sessions: the first on chlamydia, contraception and condoms and the second on HIV. All general practices in each participating LA were offered training and those that accepted were randomly allocated to receive the first training session in one of three phases as part of the step-wedge design of the pilot. These started in August 2013, November 2013 and February 2014. The step-wedge design allowed trainers to stagger the educational sessions while still enabling seasonal variations in chlamydia testing to be accounted for in the analysis [31]. If necessary, practices could choose to receive the training in a different phase. Practices could also choose to take part after training had already begun in other practices. Data on general practice recruitment, retention and uptake of the two training sessions were collected throughout the pilot by trainers. Feedback from participants on the training was collected using a standardised questionnaire directly after each session. Results for the 3Cs training session are presented here.

### Data sources and study outcome

Practice specific chlamydia testing and diagnosis data for patients aged 15–24 from January 2013 to September 2014 were extracted from the national chlamydia surveillance system, the Chlamydia Testing Activity Dataset (CTAD) to calculate crude chlamydia testing and diagnosis rates for the control and intervention periods. This time period diverts from the original protocol, which specified using data from February 2012 to July 2015, due to incomplete CTAD data for 2012 and October 2014 to July 2015. A follow up analysis of the longer term effect of the intervention will include data up to July 2015, when complete data is available. These data, combined with descriptive information on the practices, were used to assess changes in absolute and relative testing and chlamydia diagnosis rates per 100 registered 15–24 year old patients within each participating practice pre-intervention (control) and intervention periods.

### Statistical analysis

**Fig. 1:**
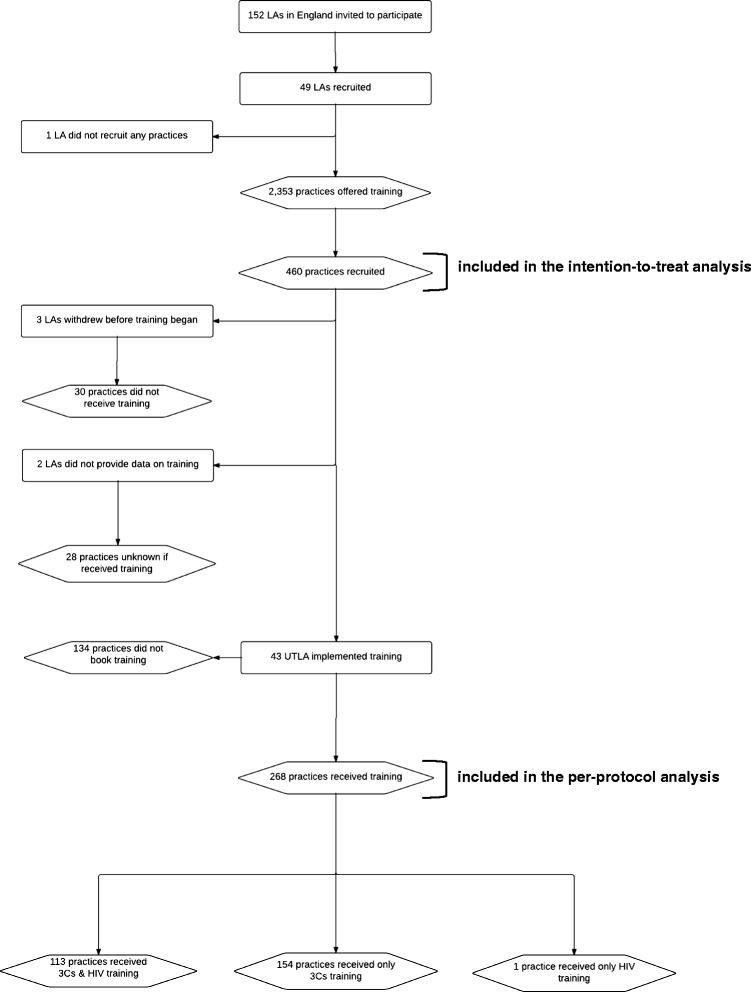
Uptake of 3Cs&HIV training by general practices across local authorities (LAs) in England

**Fig. 2:**
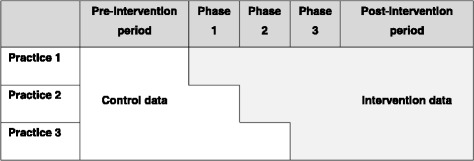
3Cs&HIV pilot step-wedge design indicating the control and intervention periods used for the analysis

Multivariable negative binomial regression models with general practice fitted as a random effect were used to estimate incident rate ratios (IRR) comparing testing and diagnosis rates pre- and post-training. Analyses were repeated using data for all practices that initially agreed to participate in the pilot (intention-to-treat (ITT)) [32] and using data for practices that received at least one training session (per-protocol (PP)) (Fig. 1). For the ITT and PP analysis, the intervention period began in the month the practice received training. For the ITT analysis only, the intervention period for the practices that agreed to participate but did not receive training, was defined as the first month of the phase the practice was allocated. Due to the step-wedge design, the number of months contributing to the control and intervention periods differed depending on which phase the general practice was allocated to and when the practice received training (Fig. 2). ITT and PP analyses were performed on all study patients, and for men and women separately. Month of test and practice population size were included as fixed effects in the model to adjust for seasonal trends in chlamydia testing and differences in practice size.

To assess effect modification the following interaction terms were included in the model using a forward step-wise approach and likelihood ratio test to decide if they should remain in this model: historical practice chlamydia testing rate per 100 15–24 year old registered population compared to national median per practice (2.6 tests per 100 patients registered); practice specific indices of multiple deprivation (IMD) (a weighted measure of deprivation using a combination of 38 separate indicators) [33]; number of general practitioners (GPs) employed per practice, number of nurses employed per practice; whether the practice is paid specifically for chlamydia testing before receiving training; practice urban/rurality classification; GUM clinic proximity to practice; phase of implementation and practice LA. Two additional variables were included in the PP models to assess fidelity: the number of GPs and nurses who attended the first training session.

All analyses were carried out using STATA v13.

## Results

### Process evaluation: uptake of intervention

One third of English LAs (49/152) chose to participate in the 3Cs&HIV pilot offering training to 29 % (2,343/8,038) of general practices in England. Of these practices, 20 % (460/2,343) agreed to participate with 512,567 patients aged 15–24 years registered. Overall 58 % (268/460) of practices that agreed to participate received at least one training session. However, only 25 % (113/460) received both the 3Cs and HIV sessions. Of those that received training, 68 % (183/268) were randomised to a phase. The remaining third were not randomised to phase as they chose to participate after the initial recruitment process. Of those that were randomly allocated to a phase, 67 % (124/183) received training in the phase allocated. Six LAs did not implement training; for one LA, no practices agreed to participate, three LAs had staffing problems and two LAs did not provide data about implementation; as a result 58 practices who agreed to participate were excluded in the per-protocol analysis along with a further 134 practices that did not receive training despite agreeing to participate. Figure 1 outlines recruitment and retention of LAs and general practices.

The practices that received training (per-protocol) were broadly similar to all recruited practices (intention-to-treat) (Table 1). Similar proportions of GPs and nurses attended training (GPs 36 %; 863/2367: nurses 40 %; 808/2011). However, when compared to the total number of GPs or nurses employed by the practice, a higher proportion of nurses attended training.

**Table 1:** Characteristics of practices participating in the 3Cs & HIV pilot

	Intention-to-treat *N* = 460 (%)	Per-protocol *N* = 268 (%)
Chlamydia testing rate below England median (<2.6 per 100)	163 (35.4)	183 (68.3)
Proximity to GUM clinic		
Within 5 km	209 (45.4)	120 (44.8)
5-10 km	117 (32.0)	63 (23.5)
Further than 10 km	134 (29.1)	85 (31.7)
Practice IMD^a^ group		
1 (most deprived)	75 (16.3)	40 (14.9)
2	85 (18.5)	51 (19.0)
3	112 (24.3)	60 (22.4)
4	87 (18.9)	51 (19.0)
5 (least deprived)	98 (21.3)	66 (24.6)
Unknown	3 (0.7)	0 (0.0)
Rural location	27 (5.9)	21 (7.8)
Urban location	433 (94.1)	247 (92.2)
Number of GPs employed		
1	12 (2.6)	6 (2.2)
2-5	130 (28.2)	74 (27.6)
6-10	158 (34.3)	98 (36.6)
11-15	108 (23.5)	58 (21.6)
16+	52 (11.3)	32 (11.9)
Number of nurses employed		
2-5	283 (61.5))	164 (61.2)
6-10	111 (24.1)	69 (25.8)
11-15	9 (2.0)	7 (2.6)
Unknown	57 (12.4)	28 (10.5)
Financial incentive for testing in place before training		
No	43 (9.3)	39 (14.6)
Yes	148 (32.2)	132 (49.2)
Unknown	269 (58.5)	97 (36.2)

^a^Indices of multiple deprivations

### Participant feedback on training

**Fig. 3:**
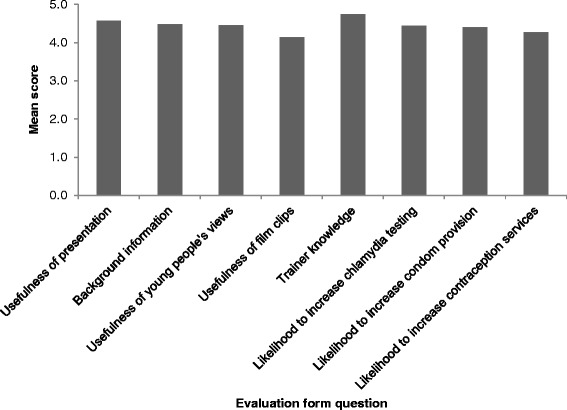
Results of the training session evaluation form completed by participants

Feedback scores were available from 1,156 evaluations forms from 121/267 3Cs training sessions. Not all trainers were able to collect feedback from all sessions because not all staff completed the questionnaire. All mean scores were above 4/5 (Fig. 3). Trainer’s knowledge and usefulness of the presentation scored highest (4.7 and 4.6, respectively). Participants also scored a mean of 4.4 for “*How likely are you to increase your chlamydia testing as a result of this training*”. The question on the usefulness of short educational videos scored lowest. Evaluation forms highlighted that there were difficulties viewing these videos in the practice setting due to the equipment available.

### Regression analysis

**Fig. 4:**
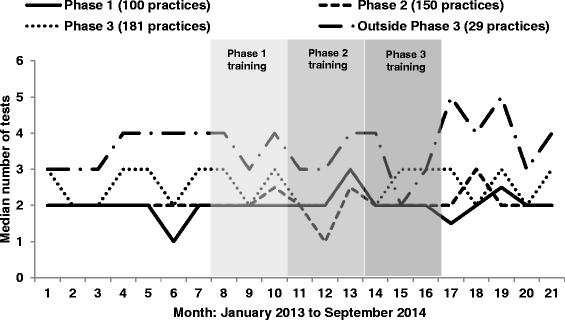
Median tests per practice per month split by phase of training started

Within all 460 practices recruited, during the pre-intervention period (control) there were 26,021 chlamydia tests completed and 18,797 in the post-intervention period. The median testing rate per practice per month was 2.7 in both the control and intervention period. In the control period 1,493 chlamydia infections were detected compared with 955 in the intervention period. The median chlamydia diagnosis rate per practice per month being 0.1 in both control intervention period. Figure 4 outlines the unadjusted median tests per month from January 2013 to September 2014 for practices allocated to each phase of the pilot. Median tests fluctuated between 1 and 3 tests per month for all practices that received training (or were allocated to receive training) in phase 1, 2 or 3. Practices that received training after phase 3 (between May 2014 and September 2014) did more tests per month, fluctuating between 2 and 5 tests.

After adjusting for variables associated with chlamydia testing, the regression analysis found no significant change in chlamydia testing or diagnosis following training (testing Incident Rate Ratio (IRR) 1.01 95 % confidence interval (CI) 0.96–1.07, *P* = 0.72; diagnosis IRR 0.98 (0.84–1.15), *P* = 0.84). However, the intervention increased testing significantly in practices that received specific payments for testing before the pilot began (IRR 2.12 (1.41–3.18), *P* < 0.001). A statistically significant effect was seen when the number of GPs employed by the practice was included as an effect modifier with practices employing between 6 and 15 GPs seeing significant increases (6–10 GPs IRR 1.35 (1.07–1.71), *P* = 0.012; 11–15 GPs IRR 1.37 (1.09–1.73), *P* = 0.007). A near significant increase in testing was observed in practices with a lower than English median testing rate prior to the intervention (IRR 1.54 (0.99–2.37), *P* = 0.051), practices that did not get paid for testing prior to the intervention (IRR 1.54 (0.99–2.37), *P* = 0.051) and practices with ≥16 GPs employed (IRR 1.27 (0.99–1.61), *P* = 0.053) (Table 2).

**Table 2:** Intention-to-treat analysis (460 practices) with adjusted stratification comparing change in chlamydia testing pre- & post-intervention

Practice characteristic & sub-group	Total number of practices	Adjusted incident rate ratio (95 % confidence interval; *P* value)
Chlamydia testing rate per practice in 2013		
Less than England median	163	1.54 (0.99-2.37; 0.051)
Greater than England median	297	1.05 (0.69-1.60; 0.823)
Payment for chlamydia screening		
Yes	148	2.12 (1.41-3.18; <0.001)
No	43	1.54 (0.99-2.37; 0.051)
Unknown	269	1.77 (1.19-2.65; 0.005)
Number of GPs employed		
1	12	1.54 (0.99-2.37; 0.051)
2-5	130	1.19 (0.94-1.51; 0.156)
6-10	158	1.35 (1.07-1.71; 0.012)
11-15	108	1.37 (1.09-1.73; 0.007)
16+	52	1.27 (0.99-1.61; 0.053)

The per-protocol analysis model found the same overall result as the intention-to-treat analysis, except no difference was found in intervention effect between practice sub-groups (see Additional file 1 for full results plus analysis of practices that received training in phase randomised to). The sub-group analyses in men and women found no evidence to suggest any difference of effect between these groups.

## Discussion

### Summary

This large national pilot found no major impact on chlamydia testing and diagnosis rates in participating general practices following implementation of 3Cs&HIV training despite staff reporting that they perceived the training as useful and that it had increased their likelihood of offering this service. There was evidence that the intervention increased testing in certain sub-groups, these being practices with financial payments already in place or with between 6 and 15 GPs. This indicates that there are remaining barriers preventing intentions to test being converted into actual tests submitted, and demonstrates the importance of fully piloting and evaluating any service improvement intervention to assess the impact before widespread implementation. Our qualitative work with GP staff who were involved in the workshops (reported separately), suggests that the intervention may not have been implemented exactly as intended. This indicates the need for developing detailed understanding of local services, and resources in order to select effective interventions.

The impact of this pilot differ from the intervention it was based on, CIRT, possibly due to several factors; changing context of the health care system with increasing pressures on GP staff since the original CIRT study; because the intervention was implemented in different parts of the country; or increased complexity from the original intervention in part due to the additional elements added to the intervention to fit in with current policy so that each key message may have been diluted or been perceived by GP staff as too onerous.

### Strengths and limitations

The major strengths of this evaluation, unlike a cluster randomised controlled trial within a research setting, are: the large number of LAs with an opportunity to be involved, the ability to ascertain the acceptance rate of the intervention by LAs and take-up rate of training by practices across a large sector of a country, and the ability to robustly evaluate the implementation of an intervention with a priori evidence of efficacy.

Evaluation of the pilot was limited by data availability; this may have restricted the ability to identify further factors which influenced effectiveness. Data on which parts of the complex intervention were implemented, the number of trainers per LA or detailed sexual health training experience of trainer were absent. Missing data for 28 practices may also explain why the sub-group impact identified in the ITT analysis was not found in the PP analysis, as these practices were not included in the PP analysis. We could not determine the number of offers of chlamydia tests by general practice staff, which was also a limitation of other general practice studies [25]. The analysis was limited by the quality of surveillance data to measure tests and diagnoses per practice. We could not determine the tests and diagnoses by individual staff attending training, only per practice. Allowing practices to change the phase they are randomised to for training has the potential to introduce bias, as they represent a motivated group. It’s unclear what the direction of this bias would be as high screening practices saw minimal effect. The lack of sub-group impact found in the ITT analysis in the PP analysis may be due to missing data from practices that were not included

### Comparison with existing literature & implications for further research

Although not effective for all practices, testing did increase in practices with existing financial incentives. Combining financial rewards with educational training has proven effective in other countries [26]. However, in England, education and peer support have proven more effective than financial incentives alone [34]. The 3Cs&HIV has shown that practices with existing financial incentives are more susceptible to training interventions than those who have none. It is important to recognise that the 3Cs&HIV training did not introduce any new funding for testing and only sought to remind practices of incentives already in place.

Following CIRT, and in line with other interventions [25], 3Cs&HIV training emphasises the inclusion of all general practice staff in training [20, 35]. The intervention effect differed depending on the number of GPs employed but not nurses. The difference between the number of GPs having an effect and the number of nurses could be a measure of how influential GPs are within a practice. Alternatively higher GP numbers could be a marker of sufficient capacity for each practitioner to follow their special interest such as sexual health, or increased time for staff for public health interventions.

Testing increased to near significant levels in practices doing very little or no chlamydia testing prior to the intervention, suggesting 3Cs&HIV training could be used as a starter programme alongside more intensive training programmes for practices that have limited screening [36]. The absence of effect in practices with above average testing rates prior to the intervention may reflect interested practices taking up the training but who already test at full capacity.

Despite the significant increases seen in testing rates in some sub-groups, it’s important to recognise that the number of tests and diagnoses per practice is still low following training and these increases will not have a widespread impact on national chlamydia testing rates or detection rates to meet the Public Health Outcomes Framework indicator of 2,300 diagnoses per 100,000 15 to 24 year olds in each LA [37].

This evaluation shows the difficulties in maintaining practice engagement with sexual health training even with those who have identified this as a training need and volunteered to take part, as demonstrated by high practice drop-out through the course of the pilot. Trainers commonly found arranging sessions with practices time consuming. General practice drop out of pilots or studies is not uncommon and has been identified in other recent studies in England [38–40]. Future primary care studies should carefully identify which stakeholders influence service provision in general practices and engage with these organisations early on. This pilot worked with LAs as they are responsible for commissioning public health services from primary care. However, engagement with the Local Medical Committee and Clinical Commissioning Group may be equally as important.

## Conclusions

3Cs&HIV training is just one method aimed at improving sexual health services in primary care. Improving chlamydia testing provision in general practices remains important. Over 70 % of young people attend their general practice in a year and evidence suggests that young people want practitioners and professionals to offer chlamydia testing rather than them having to ask for it [6, 9]. Other methods for general practice engagement to convert intention to test into actual increased testing rates should continue to be explored and rigorously evaluated with local support before being endorsed by public health bodies.

Based on these findings, the authors do not endorse adoption of the current 3Cs&HIV training as delivered in this study, as a method of increasing chlamydia screening or detection rates. The decision to use the 3Cs&HIV training materials needs to be made locally; local teams should take into consideration the specific practices in their area, the resources available to them to ensure training is completed as intended, and how screening in general practice fits with wider strategies.

## Abbreviations

3Cs& HIV, chlamydia testing, contraception and condom provision & HIV testing; CI, 95 % confidence interval; CIRT, chlamydia intervention randomised trial; CTAD, chlamydia testing activity dataset; GP, general practitioner; GUM, genitourinary medicine clinic; IMD, indices of multiple deprivation; IRR, incident rate ratio; ITT, intention-to-treat analysis; LA, local authority; PHE, Public Health England; PP, per-protocol analysis; STI, sexually transmitted infection

## Additional file

Additional file 1: Contains the per-protocol analysis for the 268 practices that received at least one training session. (DOC 37 kb)
