# Supplementary material for: Service evaluation of an educational intervention to improve sexual health services in primary care implemented using a step-wedge design: analysis of chlamydia testing and diagnosis rate changes
Source: BMC Public Health. 2016 Aug 2;16:686. doi: 10.1186/s12889-016-3343-z (PMC4969638; doi:10.1186/s12889-016-3343-z)
Supplement: Additional file 1: — Contains the per-protocol analysis for the 268 practices that received at least one training session. (DOC 37 kb) [file 12889_2016_3343_MOESM1_ESM.doc]

**Service evaluation of an educational intervention to improve sexual health services in primary care implemented using a step-wedge design: analysis of chlamydia testing and diagnosis rate changes**

**Town et al.**

**Online supplementary material: per-protocol analysis**

The per-protocol analysis includes results from 268 practices that received at least one training session in the 3Cs & HIV pilot. After adjusting for variables associated with chlamydia testing, the multivariable negative binomial regression analysis found no significant change in chlamydia testing or diagnosis following training (testing Incident Rate Ratio (IRR) 1.00 (95% confidence interval 0.94-1.07), P=0.89; diagnosis IRR 1.01 (0.84-1.22), P=0.89).

In comparison to the intention-to-treat analysis, the intervention did not increase testing significantly in sub-groups of practices (Table 1). A near significant decrease in testing was observed in practices with between 2-5 General Practitioners (GPs) employed (IRR 0.71 (0.50-1.01), P=0.054) and 16+ GPs (IRR 0.7 (0.48-1.00), P=0.05).

Not all practices were randomised to a phase for training or received training in the phase they were allocated to. As an additional sensitivity analysis, we repeated the analysis to assess whether there was an increase in chlamydia testing in the 124 practices that received training in the phase they were randomised to. After adjusting for variables associated with chlamydia testing, the multivariable negative binomial regression analysis found no significant change in chlamydia testing or diagnosis following training (IRR 1.04 (0.94-1.16), P=0.46; diagnosis IRR 0.94 (0.67-1.32) P=0.723).

**Table 1: Per-protocol analysis (268 practices) with adjusted stratification** comparing change in chlamydia testing pre- & post-intervention

| **Practice characteristic & sub-group** | **Total number of practices** | **Adjusted incident rate ratio**  **(95% confidence interval; P value)** |
| --- | --- | --- |
| **Chlamydia testing rate per practice in 2013** | | |
| Less than England median | 183 | 1.04 (0.42-2.55; 0.937) |
| Greater than England median | 85 | 0.69 (0.28-1.68; 0.410) |
| **Payment for chlamydia screening** | | |
| Yes | 132 | 1.38 (0.57-3.30; 0.473) |
| No | 39 | 1.04 (0.42-2.55; 0.937) |
| Unknown | 97 | 1.20 (0.50-2.86; 0.679) |
| **Number of GPs employed** |  |  |
| 1 | 6 | 1.04 (0.42-2.55; 0.937) |
| 2-5 | 74 | 0.71 (0.50-1.01; 0.054) |
| 6-10 | 98 | 0.82 (0.58-1.16; 0.255) |
| 11-15 | 58 | 0.84 (0.58-1.20; 0.331) |
| 16+ | 32 | 0.70 (0.48-1.0; 0.050) |
